# The behavior of residual tumors following incomplete surgical resection for vestibular schwannomas

**DOI:** 10.1038/s41598-021-84319-1

**Published:** 2021-02-25

**Authors:** Hun Ho Park, So Hee Park, Hyeong-Cheol Oh, Hyun-Ho Jung, Jong Hee Chang, Kyu-Sung Lee, Won Seok Chang, Chang-Ki Hong

**Affiliations:** 1 Department of Neurosurgery, Gangnam Severance Hospital, Yonsei University Health System, Seoul, Republic of Korea; 2 Department of Neurosurgery, Severance Hospital, Yonsei University Health System, Seoul, Republic of Korea; 3 Gamma Knife CenterSeverance Hospital, Yonsei University Health System, Seoul, Republic of Korea; 4 Brain Tumor Center, Severance Hospital, Yonsei University Health System, Seoul, Republic of Korea; 5 Brain Research Institute, Severance Hospital, Yonsei University Health System, Seoul, Republic of Korea

**Keywords:** Neuroscience, Diseases of the nervous system

## Abstract

The management of vestibular schwannoma (VS) with residual tumor following incomplete resection remains controversial and little is known regarding postoperative tumor volume changes. The behavior of residual tumors was analyzed for 111 patients who underwent surgery for newly diagnosed VS between September 2006 and July 2017. The postoperative tumor volume changes were assessed during a mean follow-up of 69 months (range 36–147 months). Fifty-three patients underwent imaging surveillance following incomplete resection. There was no residual tumor growth in 44 patients (83%). A significant regression of residual tumor volume was noted in the no growth group at postoperative 1 year (p = 0.028), 2 years (p = 0.012), but not from 3 years onwards. Significant predictors of regrowth were immediate postoperative tumor volume ≥ 0.7 cm^3^ (HR 10.5, p = 0.020) and residual tumor location other than the internal auditory canal (IAC) (HR 6.2, p = 0.026). The mean time to regrowth was 33 months (range 5–127 months). The 2-, 5-, and 10-year regrowth-free survival rates were 90.6%, 86.8%, and 83%, respectively. In conclusion, significant residual tumor regression could occur within 2 years for a VS with an immediate postoperative tumor volume less than 0.7 cm^3^ or residual tumor in IAC.

## Introduction

The goal of vestibular schwannoma (VS) treatment is to achieve long-term tumor control and preserve neurological function. Treatment modalities include conservative management, microsurgery, radiosurgery or combination of both micro- and radiosurgery. Surgical resection remains the primary treatment that can provide the greatest chance of a long-term cure, especially for large, symptomatic tumors^1^. However, the narrow surgical corridor of the cerebellopontine angle and the intimate relationship of VS with delicate neurovascular structures such as the facial nerve have resulted in 30–50% rates of facial nerve dysfunction with complete resection^2–5^. Recent studies have shown that a planned incomplete resection followed by upfront gamma knife radiosurgery (GKRS) could achieve both satisfactory tumor control and neurological outcome^6–9^. Unfortunately, incomplete resection carries the risks of tumor regrowth and higher radiation-dose to reduce the risks of regrowth that could lead to tumor swelling, cerebral edema, and cranial nerve neuropathy^9–13^. The inherent, additional surgical risks must also be taken into account should GKRS fail to control the tumor^14^. The question of when and under what circumstances should GKRS be performed following incomplete resection will depend on the incidence and the rate of tumor regrowth, and the benefit on neurological outcome. Several studies in the past have focused on the long-term natural history of VS, but little is known about the long-term behavior of residual VS following incomplete surgical resection. In this study, we report a long-term analysis of patients who underwent surgery for newly diagnosed VS. We analyze the behavior of residual tumors after incomplete resection by measuring the sequential postoperative tumor volume changes. We also assess the neurological outcome, especially the facial nerve function. The purpose of the study is not to support one management approach over another, but to better understand the behavior of residual VS following incomplete resection.

## Methods

A long-term, retrospective, and consecutive analysis was performed on 111 patients who underwent surgical resection for newly diagnosed VS from September 2006 to July 2017. The data of 53 patients who had residual tumor following incomplete surgical resection were put on imaging surveillance for the residual tumor. Patients with radiologic and clinical follow-up of 3 years or longer matched the inclusion criteria of this study. There were no lost to follow-up among patients who had residual tumor following incomplete resection. The decision to observe rather than to undergo an upfront GKRS was made depending on the residual tumor volume, functional outcome, and patient’s preference after informed consent. The data was gathered and analyzed after obtaining informed consent from every patient. The study was approved by the Institutional Review Board of Gangnam Severance Hospital Human Research Protection Center and conducted in accordance with the ethical guidelines of the Declaration of Helsinki.

### Data interpretation

All patients underwent magnetic resonance imaging (MRI) including T2-weighted and gadolinium enhanced T1-weighted axial, coronal, and sagittal images, acquired at 1 mm slice thickness. A scheduled imaging surveillance protocol with gadolinium enhanced MRI was performed for 15 years from the day of the surgery. The imaging studies were obtained before surgery, 3 months after surgery (immediate), annually for the first 3 years, and every 2 to 3 years thereafter. Pre- and postoperative tumor volumes were assessed by two neurosurgeons using Leksell GammaPlan software version 8.0 (Elekta AB). All performed images were also interpreted by two neuroradiologists who were blinded to patient information. Gross-total resection (GTR) was defined as complete tumor removal confirmed by the surgeon intraoperatively with no evidence of residual tumor on immediate and 1 year postoperative MRIs. Near-total resection (NTR) was defined as above 95% tumor resection with a thin layer of residual tumor on a neurovascular structure, confirmed by the surgeon intraoperatively, and on immediate and 1 year postoperative MRIs. Subtotal resection (STR) and partial resection (PR) were defined as 90–95% and less than 90% tumor resection, confirmed by the surgeon intraoperatively, and on immediate and 1 year postoperative MRIs, respectively. Tumor regrowth was defined as residual, enhancing, solid tumor volume increase of more than 20% compared to the first postoperative MRI with persistent growth on consecutive scans^15^. Neurological outcome was assessed using House–Brackmann (HB) classification for facial and Gardner–Robertson Hearing Scale (GR) for cochlear nerve function.

### Surgical procedure

The choice of surgical approach depended on the size, location, surgical line-of-sight, extent of the tumor, and surgeon’s preference. The facial nerve was routinely monitored and localized using facial nerve electromyography and stimulator throughout the surgical procedure. The cochlear nerve was monitored using intraoperative brainstem auditory evoke potentials if the patient had serviceable hearing. The cisterna magna was opened to relieve intracranial pressure and minimize cerebellar retraction. The roof of the internal auditory canal (IAC) was drilled to remove the tumor and decompress the canal to avoid retraction injury to the nerves before intracapsular debulking was performed with an ultrasonic aspirator. The facial nerve was first identified at the meatal segment, and then at the root entry zone to perform both antero- and retrograde dissection of the nerve. The intention of the surgery was a GTR, but an incomplete resection was performed if the tumor capsule was adherent to the facial nerve.

### Statistical analysis

Tumor characteristics, size, and volume changes were analyzed and compared between the no growth and regrowth group using Mann–Whitney *U* test. A Fischer’s exact test was used to compare categorical variables. Univariate logistic regression analysis was conducted to determine significant predictors of regrowth. A Cox proportional hazards model was used to report hazard ratios of significant factors with 95% confidence intervals. A stepwise multivariate logistic regression analysis was also performed to seek any independent predictors of regrowth. Regrowth-free survival was calculated using the Kaplan–Meier method. Data analyses were performed using SPSS software (version 18.0, SAS Institute Inc., Chicago, IL, USA). Graphs depicting long-term tumor volume changes and facial nerve outcome at last follow-up were plotted using Microsoft Excel software (version 16.0, Microsoft Inc., Redmond, WA, USA). Two-tailed and p values of ≤ 0.05 were considered statistically significant.

## Results

### Patient and tumor characteristics

**Figure 1:**
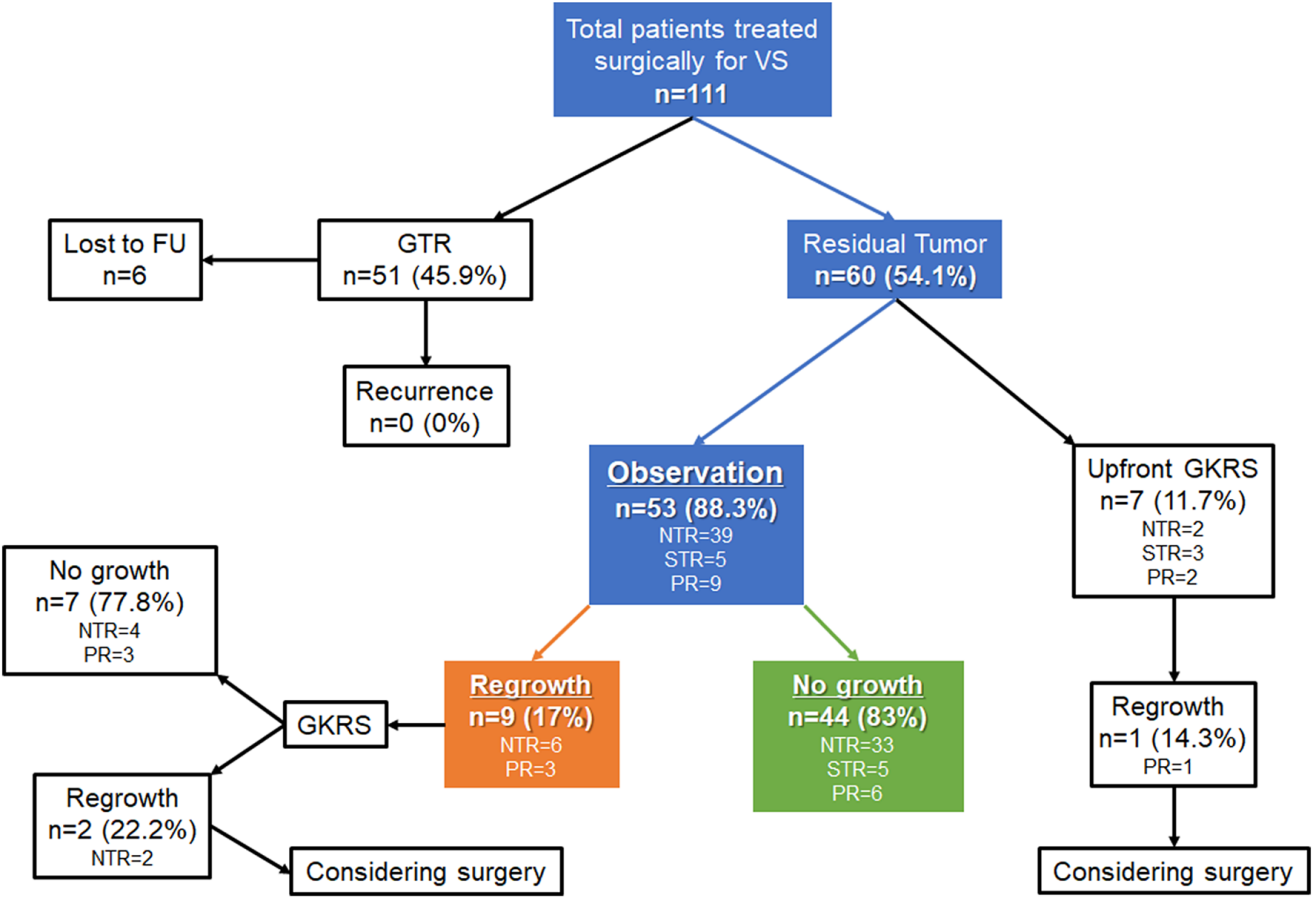
Flow chart showing the management and outcome of patients with residual tumor following incomplete resection.

One hundred and eleven patients underwent surgery for newly diagnosed VS, in which 51 (45.9%) achieved GTR with no recurrence during the study period (Fig. 1). Among 60 patients (54.1%) with residual tumor following surgical resection, 7 patients underwent upfront GKRS and 53 patients were put on imaging surveillance for the residual tumors. Of the 53 patients with incomplete resections, there were 36 female (67.9%) and 17 male (32.1%) patients with a mean age of 50 years (range 20–75 years) (Table 1). The mean tumor length, width, height, and volume were 3.4 cm (range 1.2–6.1 cm), 2.6 cm (range 0.8–4.5 cm), 2.6 cm (range 1.0–4.8 cm), and 13.5 cm^3^ (range 0.7–42.7 cm^3^), respectively. All patients had tumors that were Koos grade III (28.3%) or IV (71.7%) with a solid (64.2%) or cystic (35.8%) character. The foremost symptom was hearing difficulty (75.5%) with a GR class of I to V in 24.5%, 20.8%, 28.3%, 5.7%, and 20.8% of patients, respectively. The rate of preoperative unserviceable hearing (GR classes III to V) was 54.8%. The preoperative facial nerve function was mostly normal (94.3%) with a HB grade I. There was 1 patient (1.9%) with HB grade II and 2 (3.8%) with HB grade IV facial nerve function. The mean radiologic and clinical follow-up was 69 months (range 36–147 months). The rate of 3-, 5-, 7-, and 10-year radiologic and clinical follow-up were 100%, 52.8%, 26.4%, and 9.4%, respectively.

**Table 1:** Summary of patients with incomplete surgical resection for vestibular schwannomas.

Variable	Value
Age in years (range)	50 (20–75)
**Sex (%)**	
Male	17 (32.1)
Female	36 (67.9)
**Tumor size in cm (range)**	
Length	3.4 (1.2–6.1)
Width	2.6 (0.8–4.5)
Height	2.6 (1.0–4.8)
**Koos grade (%)**	
III	15 (28.3)
IV	38 (71.7)
**Tumor character (%)**	
Solid	34 (64.2)
Cystic	19 (35.8)
**Preoperative HB grade (%)**	
I	50 (94.3)
II	1 (1.9)
IV	2 (3.8)
Preoperative tumor volume in cm^3^ (range)	13.5 (0.7–42.7)
**Extent of resection (%)**	
NTR	39 (73.6)
STR	5 (9.4)
PR	9 (17)
Extent of resection in % (range)	95.8 (77.8–99.8)
**Postoperative tumor volume in cm**^**3**^	
Immediate	0.36 ± 0.54
1-year	0.28 ± 0.37
2-year	0.31 ± 0.51
3-year	0.35 ± 0.55
5-year	0.54 ± 1.11
7-year	1.01 ± 2.08
10-year	0.20 ± 0.22
**Site of residual tumor (%)**	
IAC	44 (83)
BS	3 (5.7)
CN5	3 (5.7)
FN + BS	2 (3.8)
FN	1 (1.9)
**Postoperative HB grade (%)**	
I	27 (50.9)
II	14 (26.4)
III	9 (17)
IV	2 (3.8)
V	1 (1.9)
**Surgical morbidity (%)**	
None	48 (90.6)
Facial numbness	3 (5.7)
Diplopia	2 (3.8)
Regrowth (%)	9 (17)
Surgery to regrowth time in months (range)	33 (5–127)
Follow-up time in months (range)	69 (36–147)

*BS* brain stem, *CN5* trigeminal nerve, *FN* facial nerve, *HB* House–Brackmann, *IAC* internal auditory canal, *NTR* near-total resection, *PR* partial resection, *STR* subtotal resection.

*Values shown are mean.

### Tumor control and radiologic outcome

**Figure 2:**
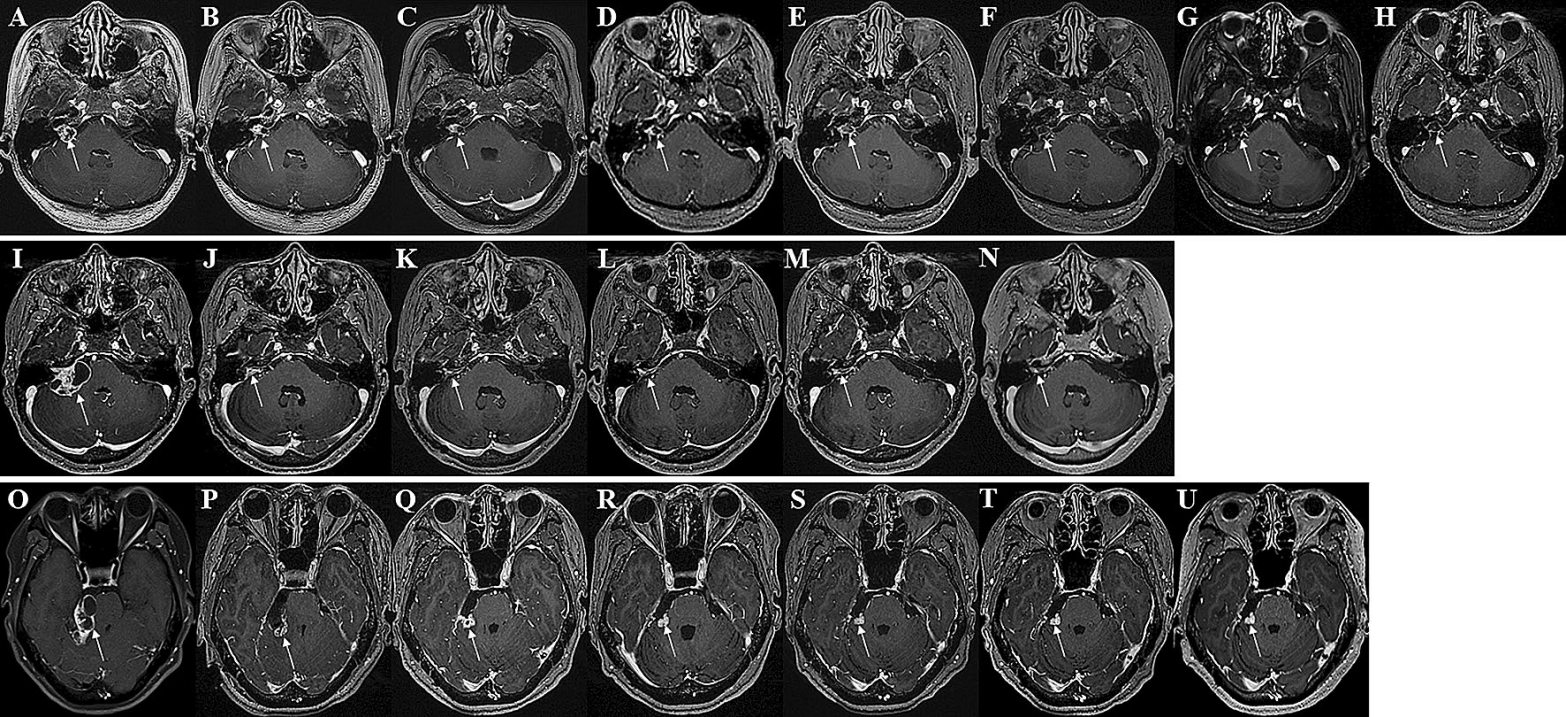
Preoperative (**A**,**I**,**O**), immediate (**B**,**J**,**P**), 1- (**C**,**K**,**Q**), 2- (**D**,**L**,**R**), 3- (**E**,**M**,**S**), 5- (**F**,**N**,**T**), 7- (**G**,**U**), and 10-year (**H**) postoperative gadolinium enhanced T1-weighted axial MR images of patients with residual tumor (white arrows) following incomplete resection. (**A**–**H**) The residual tumor in IAC of a patient with partial resection (85.2% extent of resection) gradually regressed from an immediate postoperative tumor volume of 1.19 cm^3^ (**B**) to 0.01 cm^3^ (**H**) over a follow-up of 147 months. The patient had a normal facial nerve function at last follow-up. (**I**–**N**) The residual tumor in IAC of a patient with subtotal resection (93% extent of resection) gradually regressed from an immediate postoperative tumor volume of 0.62 cm^3^ (**J**) to 0.2 cm^3^ (**N**) over a follow-up of 62 months. The patient had a normal facial nerve function at last follow-up. (**O**–**U**) The residual tumor at the brain stem of a patient with near-total resection (96.2% extent of resection) gradually regressed from an immediate postoperative tumor volume of 0.67 cm^3^ (**P**) to 0.38 cm^3^ (**U**) over a follow-up of 84 months. The patient had a facial nerve function of HB grade II at last follow-up.

**Figure 3:**
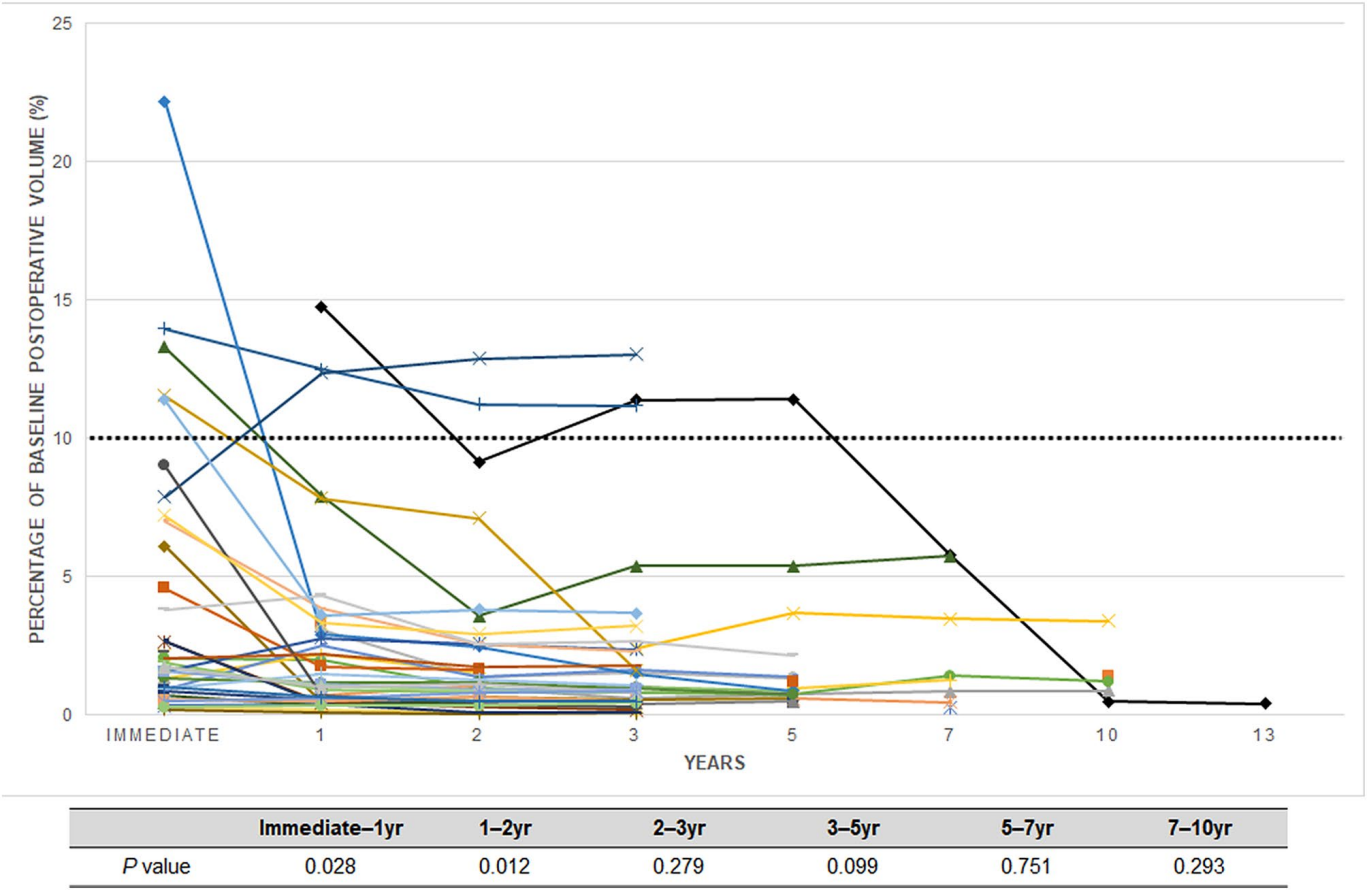
Graph outlining long-term tumor volume changes of patients with no residual tumor growth after incomplete resection. The y axis indicates the percentage of residual tumor volume compared to the preoperative tumor volume. There was significant reduction of tumor volume compared to the prior MRI scan at postoperative 1 year (p = 0.028) and 2 years (p = 0.012), but not from 3 years (p = 0.279) onwards.

**Figure 4:**
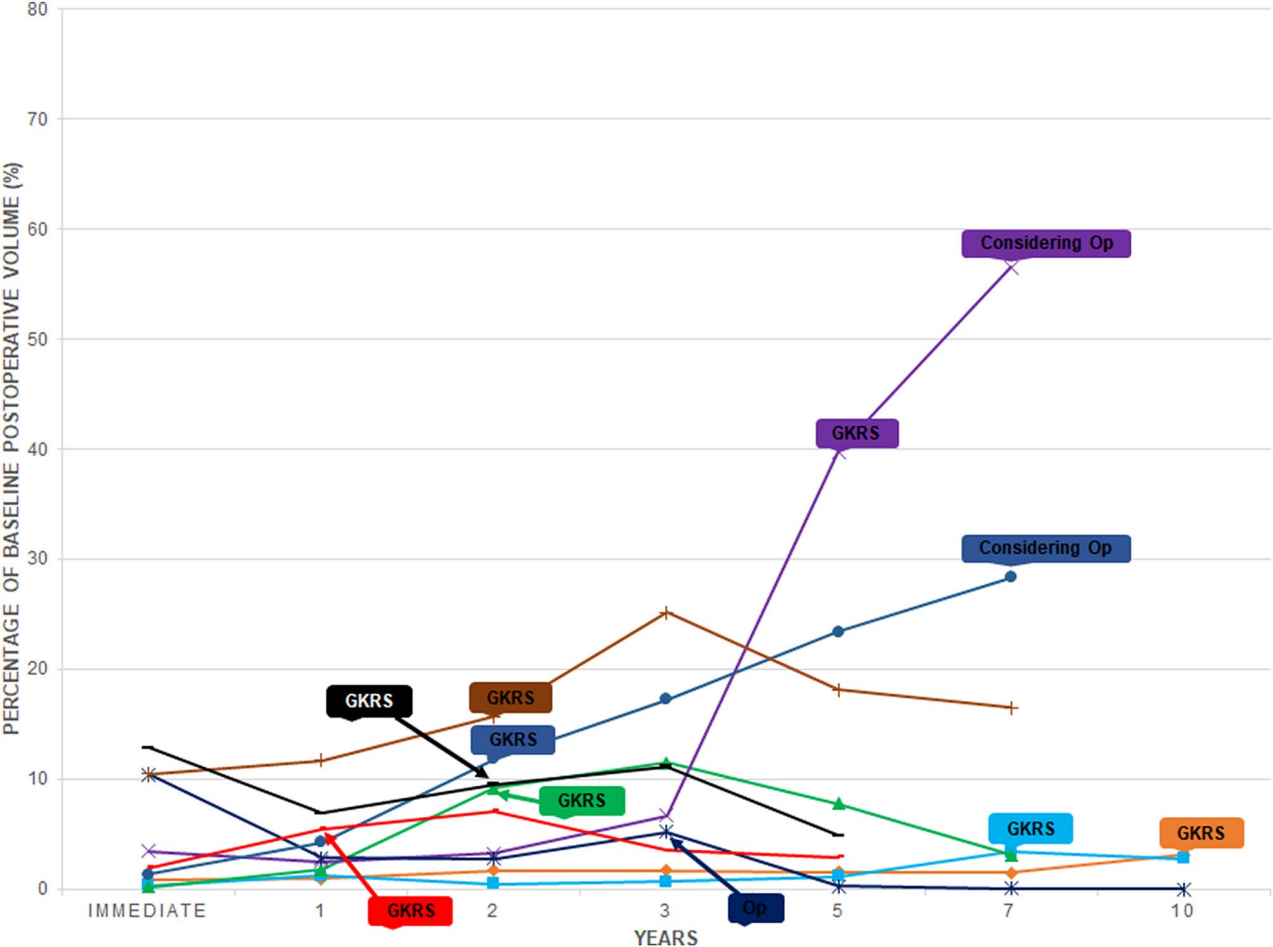
Graph outlining long-term tumor volume changes of patients with residual tumor growth after incomplete resection. The y axis indicates the percentage of residual tumor volume compared to the preoperative tumor volume. The mean time from surgery to residual tumor growth was 33 months (range 5–127 months). There were two patients with a late regrowth at postoperative 76 and 127 months. Among patients with residual tumor growth, 8 patients underwent salvage GKRS and 1 patient underwent salvage surgical resection. There were 2 patients (22.2%) with persistent residual tumor growth after GKRS and are under consideration for a salvage surgical resection.

**Figure 5:**
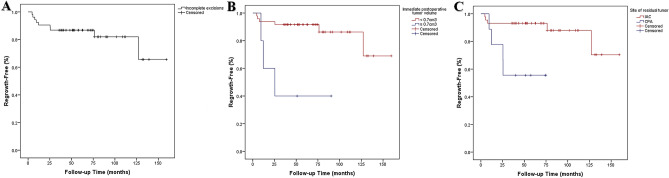
Kaplan–Meier curves of regrowth-free period following incomplete resection (**A**) and dichotomized for immediate postoperative tumor volume (**B**), and site of residual tumor (**C**).

Retrosigmoid lateral suboccipital (92.5%) and presigmoid retrolabyrinthine posterior transpetrosal (7.5%) approaches were performed for 53 patients with incomplete resections. NTR was achieved in 39 patients (73.6%), STR in 5 patients (9.4%), and PR in 9 patients (17%). The mean extent of resection (EOR) for incomplete resections was 95.8% (range 77.8–99.8%) with a mean immediate postoperative tumor volume of 0.36 cm^3^ (range 0.01–3.64 cm^3^). The most common site of residual tumor was IAC (83%), followed by the brain stem (5.7%), trigeminal nerve (5.7%), brain stem and facial nerve (3.8%), and facial nerve (1.9%) (Fig. 2). There was no residual tumor growth in 44 patients (83%) and regrowth in 9 patients (17%) during the study period. There was significant reduction of tumor volume compared to the prior MRI scan in the no growth group at postoperative 1 year (p = 0.028) and 2 years (p = 0.012), but not from 3 years (p = 0.279) onwards (Fig. 3). The mean time from surgery to residual tumor growth in the regrowth group was 33 months (range 5–127 months), in which 8 patients underwent salvage GKRS and 1 patient underwent surgical resection (Fig. 4). The were 2 patients (22.2%) with persistent residual tumor growth after GKRS. The mean regrowth-free period for VS with incomplete resections was 131 months (95% CI 114–148 months). The regrowth-free survival at 2, 5, and 10 years was 90.6%, 86.8%, and 83%, respectively (Fig. 5A).

### Predictors of tumor regrowth

There were no differences in patient and tumor characteristics such as age, sex, preoperative tumor size and volume, and Koos grade between the no growth and regrowth group (Table 2). The mean EOR of no growth and regrowth group was 96% and 95.2%, respectively (p = 0.903). The mean preoperative, immediate postoperative, 1-, 2-, 3-, 5-, 7-, 10-, and 13-year tumor volumes were 12.2 cm^3^, 0.27 cm^3^, 0.19 cm^3^, 0.14 cm^3^, 0.14 cm^3^, 0.12 cm^3^, 0.08 cm^3^, and 0.11 cm^3^ in the no growth and 18.5 cm^3^, 0.8 cm^3^, 0.69 cm^3^, 1.04 cm^3^, 1.21 cm^3^, 1.41 cm^3^, 1.95 cm^3^, and 0.35 cm^3^ in the regrowth group, respectively. On univariate analysis, significant predictors of regrowth were immediate postoperative tumor volume ≥ 0.7 cm^3^ (HR 10.5, 95% CI 1.445–76.289, p = 0.020) (Fig. 5B) and residual tumor location other than IAC (HR 6.2, 95% CI 1.246–31.250, p = 0.026) (Fig. 5C), but no significance could be established on multivariate analysis.

**Table 2:** Characteristic differences between no growth and regrowth group following incomplete surgical resection for vestibular schwannomas.

	No growth	Regrowth	*P*
Patients	44	9	
Age (years)*	50 ± 13.6	49.7 ± 11.2	0.820
Sex, M/F	13/31	4/5	0.445
Tumor size (cm)*			
Length	3.3 ± 1.2	4 ± 1	0.064
Width	2.6 ± 0.9	2.9 ± 0.9	0.312
Height	2.5 ± 0.9	3.1 ± 0.8	0.064
**Koos grade**			NA
III	15 (34.1)	0 (0)	
IV	29 (65.9)	9 (100)	
**Tumor character**			0.706
Solid	29 (65.9)	5 (55.6)	
Cystic	15 (43.1)	4 (44.4)	
**Preoperative HB grade**			NA
I–II	42 (95.5)	9 (100)	
III–V	2 (4.5)	0 (0)	
Preoperative tumor volume (cm^3^)	12.2 ± 9.9	18.5 ± 12.3	0.128
**Extent of resection**			0.684
NTR	33 (75)	6 (66.7)	
STR + PR	11 (25)	3 (33.3)	
Extent of resection (%)*	96 ± 4.9	95.2 ± 5.2	0.903
**Immediate postoperative tumor volume (cm**^**3**^**)**			0.030
< 0.7	42 (95.5)	6 (66.7)	
≥ 0.7	2 (4.5)	3 (33.3)	
**Site of residual tumor**			0.035
IAC	39 (88.6)	5 (55.6)	
CPA	5 (11.4)	4 (44.4)	
**Postoperative HB grade**			0.665
I–II	33 (75)	8 (88.9)	
III–V	11 (25)	1 (11.1)	

*CPA* cerebello-pontine angle, *HB* House-Brackmann, *IAC* internal auditory canal, *NA* not applicable, *NTR* near-total resection, *PR* partial resection, *STR* subtotal resection.

*Values shown are mean ± SD.

### Functional outcome and surgical complications

**Figure 6:**
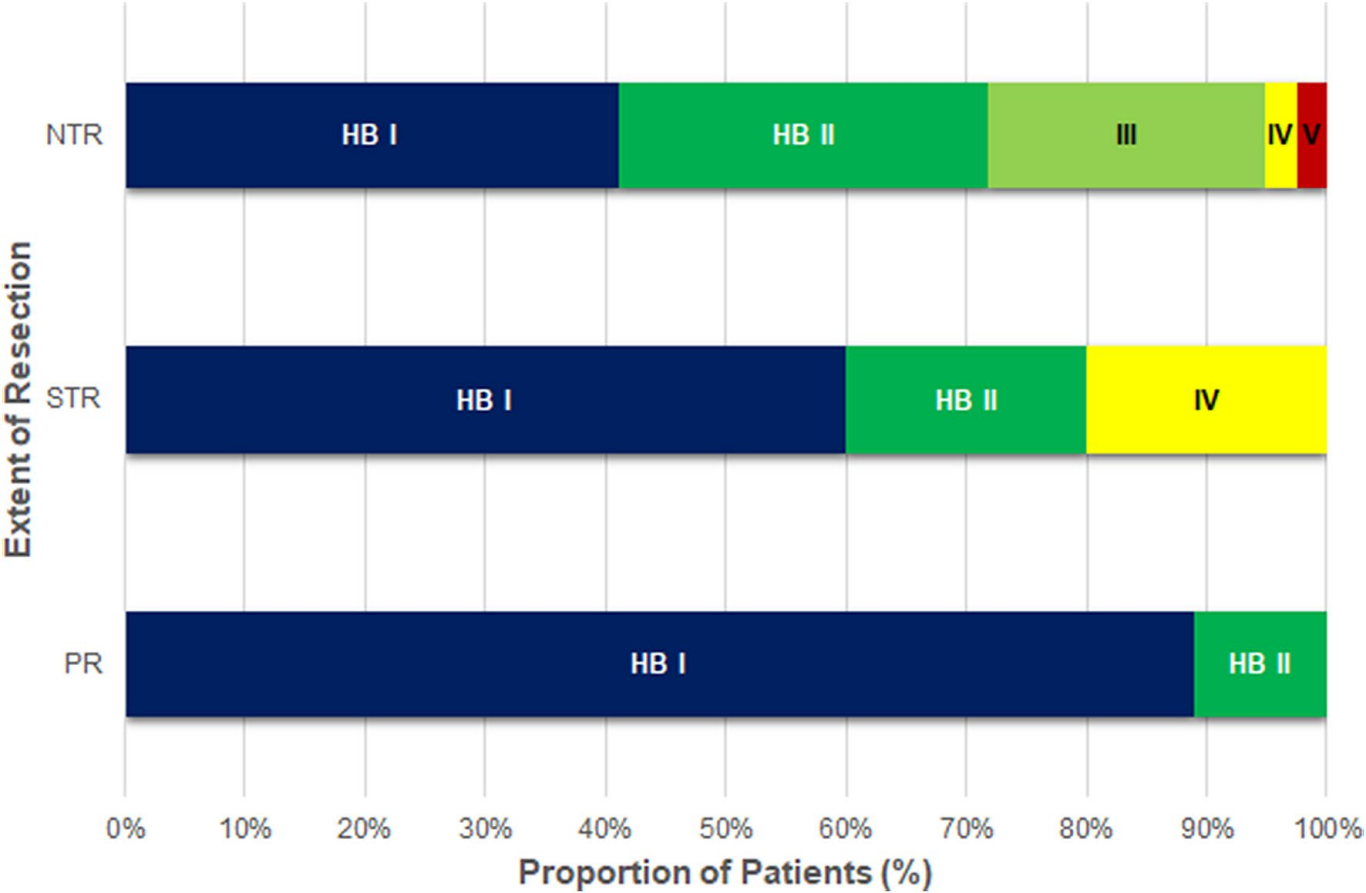
Graph summarizing facial nerve outcome at last follow-up according to extent of resection for vestibular schwannomas with incomplete resection. The rates of optimal HB (grades 1 and 2) were 71.8% for NTR, 80% for STR, and 100% for PR.

The postoperative facial nerve function at last follow-up was HB grades I to V in 50.9%, 26.4%, 17%, 3.8%, and 1.9% of patients, respectively. Compared to the facial nerve status before surgery, 50.9% of patients had completely normal facial nerve function, 3.8% had no change, 28.3% had deterioration by 1 grade, 13.2% by 2 grades, 1.9% by 3 grades, and another 1.9% by 4 grades. The rates of optimal HB (grades 1 and 2) were 71.8% for NTR, 80% for STR, and 100% for PR (Fig. 6). Postoperatively, 7.5% of patients retained serviceable hearing, unserviceable hearing remained the same in 54.8%, and 37.7% had deterioration. There were no differences in postoperative facial nerve function and hearing between the no growth and regrowth group. As for surgical morbidities, 5 of 44 patients (11.4%) in the no growth group had neurological deterioration. Three patients with facial numbness had near-complete recovery at last follow-up and the diplopia of 2 patients could be corrected with prism glasses. There were no surgical morbidities in the regrowth group.

## Discussion

The management of large VSs has evolved over the last 30 years based on long-term tumor control and preservation of neurological function. Historically, a complete surgical resection carried the least risk of a long-term recurrence, but came at a 30–50% risk of facial nerve dysfunction^2–5,16^. The concern that an aggressive surgical resection would cause inadvertent trauma to the facial nerve and other critical neurovascular structures has recently brought a shift in surgical practice. With advances in radiosurgical techniques, a planned incomplete resection followed by upfront GKRS has become a feasible treatment strategy that could achieve both satisfactory tumor control and neurological outcome^6–9,17^. However, an incomplete resection with upfront GKRS is not completely free from risks of its own such as pseudoprogression, cerebral edema, radiation injury to the brain stem, cranial nerve neuropathy, malignant transformation, and added surgical risks should GKRS fail^9–14^. Therefore, it is important to understand the behavior of residual tumors following an incomplete resection. Some authors showed that small, devascularized residual tumors do not grow^18–20^, while others proved that the proliferative activity of the tumor itself contributes to regrowth^21–23^. The question of whether the residual tumor will grow or not will depend on the pattern of regrowth, in which the incidence and rate of regrowth, and the benefit on neurological outcome need to be addressed. So far, the long-term behavior of residual tumors and the clinicopathologic factors that contribute to regrowth are not well understood. Therefore, we explored the long-term behavior of residual VS following incomplete resection, in terms of sequential tumor volume changes and assess the neurological outcome.

### Definition of regrowth and extent of resection

The definition of two terminologies, regrowth and EOR has to be clarified before assessing the long-term behavior of residual tumors and the factors that affect them. There is currently no gold standard to define the growth of residual tumor in literature. Hence, many authors have traditionally determined regrowth as tumor size increase in its greatest dimension on follow-up MRIs. This could be imprecise if 2-dimensional diametric measurements are used since a true residual tumor size is difficult to compare from one examination to another, especially if the tumor is small. In this study, tumor regrowth was defined as residual, enhancing, solid tumor volume increase of more than 20% compared to the first postoperative MRI scan with persistent growth on consecutive scans^7–9,15^. Postoperative leptomeningeal and perineural remodeling can be difficult to distinguish from a true residual tumor or regrowth in early postoperative MRI scans^15,24,25^, therefore the first postoperative scan was performed at postoperative 3 months with 3-dimensional volumetric determination for every scan. The same principles were applied to determine EOR by measuring the volume of residual tumor on postoperative 3 month MRI^24,25^. The surgeon’s subjective intraoperative observation of EOR was also added to our definition considering the fact that not all residual tumors appear on early postoperative MRI scans, especially after NTR.

### Behavior of residual tumors

The reported incidence of regrowth in large series that observed the behavior of residual VS after incomplete resection ranged from 6 to 21%, which was consistent with 17% of this study (Table 3). The reported mean interval between surgery and regrowth was 33–47 months with a range between 7.2 and 108 months compared to 33 months (range 5–127 months) of the present series^17,26–33^. Considering the benign, slow-growing nature of VS, a long-term radiologic follow-up is necessary to validate the rate and interval of incidence. The present series underwent a scheduled imaging surveillance protocol for a mean 69 months (range 36–147 months). No patients were lost to follow-up with a 3-, 5-, 7-, and 10-year radiologic follow-up of 100%, 52.8%, 26.4%, and 9.4%, respectively. The patients with prior micro- or radiosurgery were excluded from the analysis to understand the true behavior of residual VS following incomplete resection. According to the long-term observation of the present and other studies, patients with incomplete resections are subjected to an imaging surveillance for at least 10 years with a regrowth peak around 3 years after surgery. During the observation period, residual tumors that did not grow had significant regression of tumor volume compared to the prior MRI scan at postoperative 1 year (p = 0.028) and 2 years (p = 0.012), but not from 3 years (p = 0.279) onwards, which was consistent with the findings of Akinduro et al.^26^. It can be postulated that surgical devascularization could lead to significant necrosis and shrinkage of the residual tumor for the first 2 years after surgery^18–20^. Hence, an imaging surveillance period of at least 2 years without further intervention could be suggested considering the inherent risks of upfront GKRS^9–14^.

**Table 3:** Literature review of large series investigating the behavior of residual tumors following incomplete surgical resection for vestibular schwannomas.

Authors (year)	Pts (n)	Non-GTR (%)	Residual tumor volume* (cm^3^)	FN grade 1 and 2 (%)	Observation (%)	Regrowths among observation (%)	Surgery to regrowth time* (mos)	FU time* (mos)
Haque et al. (2011)	151	96/151 (64)	NA	97	96/96 (100)	20/96 (21)	47	72
Martin et al. (2012)	212	65/212 (31)	NA	55	65/65 (100)	5/65 (8)	NA	66
Carlson et al. (2012)	203	59/203 (29)	NA	NA	59/59 (100)	7/59 (12)	NA	42
Chen et al. (2014)	111	111/111 (100)	NA	49	111/111(100)	7/111 (6)	41	45
Monfared et al. (2016)	73	61/73 (84)	1.1	81	61/61 (100)	13/61 (21)	35	38
Syed et al. (2016)	450	42/450 (9)	NA	59	42/42 (100)	3/42 (7)	NA	73
Troude et al. (2018)	159	143/159 (90)	0.56	84	66/143 (46)	12/66 (18)	37	59
Present study	111	60/111 (54)	0.36	77	53/60 (88)	9/53 (17)	33	69

*FN* facial nerve, *FU* follow-up, *GTR* gross-total resection, *NA* not applicable/available.

*Values shown are mean and the percentages have been rounded up.

### Predictors of regrowth

Several factors including EOR^16,17,28^, residual tumor volume^17,25,27,34–36^, and the proliferative activity of the tumor itself^21–23^ have been reported to contribute to regrowth after incomplete resection of VS, but still, little is known about the significant clinicopathological factors^24,26,29–33^. In this study, an immediate postoperative tumor volume ≥ 0.7 cm^3^ had almost an 11-fold greater risk and residual tumor location other than IAC had a sixfold greater risk of a regrowth than tumors that were less than 0.7 cm^3^ or in IAC. Therefore, residual tumors with a tumor volume less than 0.7 cm^3^ or in IAC could be possible candidates for imaging surveillance without further intervention. At least with the data in literature so far, residual tumor volumes exceeding 2 cm^3^ are not good candidates for observation^17,25,34–36^. There was no significance of EOR on regrowth in the present series. This could be due to the higher mean EOR for incomplete surgical resections (95.8%) and lower mean immediate postoperative tumor volume (0.36 cm^3^) than other reported series^17,26–31,33,34^. The exclusion of patients with GTR in our analysis could have also contributed to the insignificance of EOR on regrowth.

### Extent of resection and functional outcome

Among patients with incomplete resections for VS, optimal facial nerve outcomes (HB grades I and II) have been reported to be 51–84% for NTR and 55–100% for STR^17,26–33^. The postoperative facial nerve function of the present study was within the range of the previous reports, whereby 71.8%, 80%, and 100% of NTR, STR, and PR had HB grades I and II, respectively. Although no statistical difference could be established in facial nerve outcome according to EOR the two factors seemed to have an inverse correlation. Nevertheless, the overall rate of no facial weakness at all (HB grade I) was lower than expected compared to other series^17,26–33^. This could be attributed to the larger preoperative tumor size and volume, and higher mean EOR than the reported series^17,26–33^. A larger tumor puts the facial nerve under tension which increases the likelihood of stretch injury and hypovascularization, resulting in poor facial nerve outcomes^37,38^. With regards to EOR, an inverse correlation between facial nerve outcome and EOR is not always true as long as care is taken to preserve the facial nerve^28,29,31^, which can also diminish as EOR increases. Surgical techniques such as IAC unroofing before tumor debulking, intracapsular tumor resection, and leaving behind any adherent tumor at the porus acousticus can minimize direct and indirect injury to the facial nerve. In the same sense, postoperative hearing will be retained as long as the anatomy and vascularity of the cochlear nerve is preserved^7–9^.

### Limitations

There are a small number of patients in each no growth and regrowth group, especially in the latter. Considering the benign, slow-growing nature of VS, regrowth is uncommon with 5- and 10-year regrowth-free survival rates reaching up to 85%. Consequently, a long term follow-up is essential to validate the incidence, rate, and interval of regrowth. The present series underwent a long-term imaging surveillance with no patients lost to follow-up. However, a longer follow-up might be needed as two of our patients had a late regrowth at postoperative 76 and 127 months. The lack of standard norm for regrowth and EOR is another difficulty when it comes to validating the results and comparing them with other published reports. In this study, a scheduled imaging protocol, 3-dimensional volumetric determination, and an immediate postoperative MRI taken at 3 months were adopted to overcome the ambiguous definition of regrowth. The same principles were applied to the definition of EOR by adding the surgeon’s subjective intraoperative observation of EOR as not all residual tumors appear on early postoperative MRI. The lack of data on the proliferative activity of VS is another weakness of this study since it has been reported to be a strong predictor of regrowth. The routine examination on the proliferative activity of the tumor had begun in the later stages of this study period and could not be included in the analysis. Finally, a comparison between observation and upfront GKRS for the residual tumors is beyond the scope of this study. The purpose of this study is not to support one management approach over another, but to share our findings of the behavior of residual VS after incomplete resection.

## Conclusion

Significant residual tumor regression could occur within 2 years for a VS with an incomplete surgical resection. Residual tumors with a tumor volume less than 0.7 cm^3^ or in IAC could be possible candidates for imaging surveillance without further intervention. Regrowth peak around 3 years after surgery and patients with incomplete resections are subjected to an imaging surveillance for at least 10 years as late regrowth can occur. The conclusion that residual tumors could be observed rather than preceded with upfront GKRS should be carefully interpreted, taking into account the incidence of tumor regrowth and the benefit on neurological outcome.
